# Identification of Peptide Mimotopes of *Trypanosoma brucei gambiense* Variant Surface Glycoproteins

**DOI:** 10.1371/journal.pntd.0001189

**Published:** 2011-06-14

**Authors:** Liesbeth Carolien Van Nieuwenhove, Stijn Rogé, Fatima Balharbi, Tessa Dieltjens, Thierry Laurent, Yves Guisez, Philippe Büscher, Veerle Lejon

**Affiliations:** 1 Department of Parasitology, Institute of Tropical Medicine, Antwerp, Belgium; 2 Department of Microbiology, Institute of Tropical Medicine, Antwerp, Belgium; 3 Research and Development Department, CorisBioConcept, Gembloux, Belgium; 4 Laboratory for Molecular Plant Physiology and Biotechnology, Department of Biology, University of Antwerp, Antwerp, Belgium; New York University School of Medicine, United States of America

## Abstract

**Background:**

The current antibody detection tests for the diagnosis of *gambiense* human African trypanosomiasis (HAT) are based on native variant surface glycoproteins (VSGs) of *Trypanosoma brucei* (*T.b.*) *gambiense*. These native VSGs are difficult to produce, and contain non-specific epitopes that may cause cross-reactions. We aimed to identify mimotopic peptides for epitopes of *T.b. gambiense* VSGs that, when produced synthetically, can replace the native proteins in antibody detection tests.

**Methodology/Principal Findings:**

PhD.-12 and PhD.-C7C phage display peptide libraries were screened with mouse monoclonal antibodies against the predominant VSGs LiTat 1.3 and LiTat 1.5 of *T.b. gambiense*. Thirty seven different peptide sequences corresponding to a linear LiTat 1.5 VSG epitope and 17 sequences corresponding to a discontinuous LiTat 1.3 VSG epitope were identified. Seventeen of 22 synthetic peptides inhibited the binding of their homologous monoclonal to VSG LiTat 1.5 or LiTat 1.3. Binding of these monoclonal antibodies to respectively six and three synthetic mimotopic peptides of LiTat 1.5 and LiTat 1.3 was significantly inhibited by HAT sera (*p*<0.05).

**Conclusions/Significance:**

We successfully identified peptides that mimic epitopes on the native trypanosomal VSGs LiTat 1.5 and LiTat 1.3. These mimotopes might have potential for the diagnosis of human African trypanosomiasis but require further evaluation and testing with a large panel of HAT positive and negative sera.

## Author Summary

The control of human African trypanosomiasis or sleeping sickness, a deadly disease in sub-Saharan Africa, mainly depends on a correct diagnosis and treatment. The aim of our study was to identify mimotopic peptides (mimotopes) that may replace the native proteins in antibody detection tests for sleeping sickness and hereby improve the diagnostic sensitivity and specificity. We selected peptide expressing phages from the PhD.-12 and PhD.-C7C phage display libraries with mouse monoclonal antibodies specific to variant surface glycoprotein (VSG) LiTat 1.3 or LiTat 1.5 of *Trypanosoma brucei gambiense*. The peptide coding genes of the selected phages were sequenced and the corresponding peptides were synthesised. Several of the synthetic peptides were confirmed as mimotopes for VSG LiTat 1.3 or LiTat 1.5 since they were able to inhibit the binding of their homologous monoclonal to the corresponding VSG. These peptides were biotinylated and their diagnostic potential was assessed with human sera. We successfully demonstrated that human sleeping sickness sera recognise some of the mimotopes of VSG LiTat 1.3 and LiTat 1.5, indicating the diagnostic potential of such peptides.

## Introduction

Human African trypanosomiasis (HAT), or sleeping sickness, is caused by the protozoan flagellar parasites *Trypanosoma brucei (T.b.) gambiense* and *T.b. rhodesiense*. The disease is transmitted by tsetse flies (*Glossina* spp.) and therefore only occurs in sub-Saharan Africa. The number of cases is currently estimated between 50 000 and 70 000 [1].

Control of *T.b. gambiense* HAT is largely based on accurate diagnosis and treatment of the human reservoir [2]. Detection of parasites in blood, lymph node aspirate or cerebrospinal fluid is laborious and insensitive, and therefore only applied on suspected HAT patients. In the absence of reliable antigen detection tests, the screening of the population at risk relies on the detection of antibodies against variant surface glycoproteins (VSGs) [2]. These immunogenic VSGs form a dense monolayer of homodimers that completely covers the surface of bloodstream trypanosomes and determines the variable antigen type (VAT) of the individual trypanosome [3]. The parasite genome contains hundreds of VSG genes and trypanosomes switch from the expression of one VSG gene to another. This antigenic variation enables the parasite population to survive the host's immune response. Each VSG monomer contains 400–500 amino acids and consists of two domains, a variable N-terminal domain with little primary sequence homology and a relatively conserved C-terminal domain. A glycosylphosphatidylinositol anchor links the C-terminal domain to the cell membrane. All N-terminal domains fold in a similar three-dimensional structure, exposing only a limited subset of, probably discontinuous, epitopes [4]–[7].

The current *T.b. gambiense* antibody detection tests are based on native VSGs from the VATs LiTat 1.3, LiTat 1.5 and LiTat 1.6 of *T.b. gambiense* [8]. These predominant VATs appear early during infection, and induce a strong and specific immune response in most patients [3]. The card agglutination test for trypanosomiasis (CATT) [9], most widely used for mass screening of populations at risk, consists of whole lyophilised trypanosomes of VAT LiTat 1.3 and has a sensitivity on whole blood of 87–98% and a specificity around 95% [2]. Although VSG LiTat 1.3 is not expressed in all endemic HAT foci [10], [11], the low sensitivity of CATT in those foci can be overcome by combining different VATs in one test, as is the case in the LATEX/*T.b. gambiense* and the ELISA/*T.b. gambiense* where the combination of VSG LiTat 1.3, 1.5 and 1.6 is used as antigen [12], [13].

The use of native VSGs as diagnostic antigens has several disadvantages. Firstly, non-specific epitopes on the native antigens may cause cross-reactions and decrease test specificity. Secondly, VSG production relies on culture of infective *T.b. gambiense* parasites in laboratory rodents and poses a risk of infection to the staff [14]. These disadvantages might be avoided if native antigens are replaced by synthetic peptides. The production of synthetic peptides is standardised, does not require laboratory animals and is without risk of infection [15].

Peptide phage display is a selection technique based on DNA recombination, resulting in the expression of foreign peptide-variants on the outer surface of phage virions. After an *in vitro* selection process based on binding affinity, called panning, the selected peptides are characterised by DNA sequencing. Phage display is a powerful tool to identify mimotopes, small peptides that mimic linear, discontinuous and/or non-protein epitopes [16]–[18]. Mimotopes with diagnostic potential have already been identified, e.g. for detection of specific antibodies for Lyme disease [19], hepatitis C [15], [20], typhoid fever [21], tuberculosis [22] and leishmaniasis [23]. Some mimotopes have been patented to become incorporated in commercially available tests, *e.g.* for neurocysticercosis [24].

In this study, we aimed to identify mimotopes for epitopes of *T. b. gambiense* VSG LiTat 1.3 and LiTat 1.5 that may replace the native proteins in antibody detection tests for sleeping sickness.

## Materials and Methods

### Ethics statement

Samples from HAT patients and endemic controls were collected within an observational study [13]. All individuals gave their written informed consent before providing serum. Permission for this study was obtained from the national ethical committee of DRC and from the ITM ethical committee, reference number 03 07 1 413.

### Anti-VSG monoclonal antibodies

Monoclonal antibodies (mAbs) H12H3 (IgG3, anti-VSG LiTat 1.5), H13F7 (IgG3, anti-VSG LiTat 1.3) and H18C11 (IgG1, anti-VSG LiTat 1.3) were generated by intraperitoneal infection of Balb/c mice with 10^6^ *T.b. gambiense* LiTat 1.3 and 10^6^ LiTat 1.5 cloned parasites. After 2 weeks, splenocytes were isolated and fused with NS0 myeloma cells [25]. Anti-VSG antibody producing hybridomas were identified by enzyme linked immunosorbent assay (ELISA) and further propagated. The antibodies were purified from culture supernatant on protein A agarose. The SBA Clonotyping™ system/HRP kit (Southern Biotech) was used for mAb isotyping.

### Coating of magnetic particles with anti-VSG mAbs

Anti-VSG mAbs were coated onto anti-mouse IgG functionalised magnetic particles (MP) (1% w/v, 0.35 µm, Estapor/Merck) at a concentration of 30 mg/g MP and stored in phosphate buffered saline (PBS, 0.01 mol/L phosphate, 0.14 mol/L NaCl, pH 7.4) containing 0.1% (w/v) bovine serum albumin (PBS-BSA). The coated MP were washed eight times with PBS containing 0.25% w/v gelatine and 0.1% v/v Tween-20 (PBSGT) and resuspended 0.25% w/v gelatine in PBS (PBSG). Successful coating of the MP was confirmed by agglutination of VSG coated latex beads (LATEX/*T.b. gambiense*) [12]. Anti-VSG mAb-free MPs were prepared by omitting the coating step.

### Selection of mimotopes by panning of phage-displayed peptides

Pannings were performed with the Ph.D.-12 (12-mer) and the Ph.D.-C7C (cyclic 7-mer) phage display libraries (New England Biolabs, NEB) through two rounds consisting of 1) a positive selection with anti-VSG mAbs coated on MP, 2) a negative selection with anti-VSG mAb-free MP and 3) phage amplification. After these two rounds a third positive selection was performed.

### Positive selection

For positive selection, 10 µL of the phage display library (for the 1^st^ positive selection) or 100 µL of amplified phage (for the 2^nd^ and 3^rd^ positive selection) were mixed overnight (ON) at 4°C with 1 mg mAb coated-MP in PBSG in a total volume of 1 mL. MP were washed ten times with PBSGT and bound phages were eluted by antigen competition (A) followed by acid elution (P). For the antigen competition, the MP were incubated for 1 h with 700 µL PBS containing 0.23 mg of corresponding VSG. After collection of the supernatant containing the eluted phages, MP were washed three times with PBSGT. The remaining bound phages were eluted with 600 µL of 0.2 mol/L glycine-HCl containing 1 mg/mL BSA (pH 2.2) and neutralised with 90 µL of Tris-HCl (1 mol/L, pH 9.1).

### Negative selection

The phages eluted from the positive selection were mixed overnight at 4°C with 1 mg of anti-VSG mAb-free MP in a total volume of 1 mL of PBSG. The phages in the supernatant were amplified.

### Amplification and purification of phages

Phages were amplified in *Escherichia (E.) coli* (strain ER2738, NEB) in lysogeny broth (LB), supplemented with tetracycline (20 mg/mL) [26]. After 4.5 h shaking at 37°C, bacteria were pelleted by centrifugation (30 min, 1811 g). The phages in the supernatant were precipitated overnight at 4°C with 25% w/v polyethylene glycol-6000 in 2.5 mol/L NaCl (PEG-NaCl). Phages were pelleted by centrifugation (45 min, 1811 g, 4°C) and resuspended in 1 mL of PBS. Residual bacteria were pelleted by centrifugation (5 min, 15700 g) and phage precipitation with PEG-NaCl was repeated for 2 to 4 hours at 4°C. After centrifugation (20 min, 15700 g, 4°C) the phage pellet was resuspended in 200 µL of PBS with 0.02% w/v NaN_3_.

After a third positive selection, phages selected through three antigen competition elutions (AAA) and three acid elutions (PPP) were titered as described below.

### Titering of phages

Phages were titered to obtain well separated, single plaques for analysis. Phages were diluted 10^3^ to 10^7^ in PBS, mixed with an *E. coli* culture and plated on agar plates containing 1 mL/L IPTG/X-gal (1.25 g isopropyl β-D-thiogalactoside, 1 g 5-bromo-4-chloro-3-indolyl-β-D-galactoside, 25 mL dimethylformamide). After the 3^rd^ positive selection 94 blue clones were picked and each clone was inoculated in 200 µL of LB in a sterile culture plate (BD Falcon™ Clear 96-well Microtest™ Plate). This plate was shaken overnight at 30°C, where after the bacteria were pelleted by 5 min centrifugation at 1312 g. The supernatant was tested in a sandwich ELISA with the homologous mAb. Based on these results, twenty phages per elution method were amplified, tested in a sandwich ELISA with all three mAbs and amplified for DNA extraction.

### Single-stranded DNA extraction and sequencing

Purification of phage DNA was performed according to the NEB manual [26]. Sequence determination was performed at the VIB Genetic Service Facility of the University of Antwerp with the −96 gIII sequencing primer, 5′-^H0^ CCC TCA TAG TTA GCG TAA CG-3′ (NEB). The obtained sequence chromatograms were read with Chromas 2.33 (Technelysium Pty Ltd). Sequence alignment was performed manually and with RELIC software [27]. We searched for discontinuous epitopes with the 3D-Epitope-Explorer (3DEX) [28] and visualised the sequences on the protein model with PyMOL pdb viewer (PyMOL Molecular Graphics System, Schrödinger, LLC).

### Sandwich ELISA with phage particles

ELISA plates (Nunc MaxiSorp™) were coated with 5 µg/mL anti-VSG mAb in PBS (100 µL/well) and incubated ON at 4°C. Plates were saturated for 1 h at rT with 350 µl PBS-Blotto (0.01 mol/L phosphate, 0.2 mol/L NaCl, 1% w/v skimmed milk powder, 0.05% w/v NaN_3_ ) and washed three times with 0.05% v/v Tween-20 in PBS (PBST) (ELx50, Bio-Tek ELISA washer). Wells were incubated for 4 h at rT with 100 µL of phage dilution in PBS-Blotto (1/3 for culture plate supernatant or 1/20 for PEG-NaCl purified phage). After three washes, horse radish peroxidase (HRP)-labelled anti-M13 pVIII mAb (GE Healthcare), diluted 1/2000 in PBST was added to the wells for 1 h at rT (100 µl/well). After another five washes, wells were incubated for 1 h at rT with 100 µL/well of 2.2′-azino-bis-(3-ethylbenzthiazoline-6-sulfonic acid) (ABTS) chromogen/substrate solution (50 mg tablet/100 mL of ABTS buffer, Roche). The plate was shaken for 10 seconds and the optical density (OD) was read at 414 nm (Labsystems Multiskan RC 351).

### Peptide synthesis

Peptide selection for synthesis (Eurogentec, Belgium) was based on strong, reproducible and specific reaction of phage clones with their homologous anti-VSG mAb in ELISA, prediction of peptide hydrophilicity and common motive groups in the sequences. The 12-mer linear peptides were synthesised at >70 or >85% purity. The C-terminus was elongated with a GGGS-CONH2 tail to mimic the GGGS-peptide spacer between the random peptide sequence and the phage protein pIII and to block the negative charge of the carboxyl terminus. The cyclic 7-mer sequences were synthesised at >90% purity. The 7-mer peptides were flanked by two cysteines, and the C-terminus was elongated with GGGS-CONH2. The biotinylated peptides were synthesised at >85% purity, with an additional lysine-biotin added to the C-terminus. All synthetic peptides were reconstituted in sterile deionised H_2_O to a concentration of 2 mg/mL.

### Inhibition ELISA with synthetic peptides and anti-VSG mAbs

The capacity of the synthetic peptides to mimic the natural VSG epitopes was assessed in an inhibition ELISA. Peptide dilution series of 200, 67, 22, 7 and 0 µg/ml or 67, 22, 7, 2 and 0 µg/ml were prepared in PBS containing 1% BSA (PBS-BSA) with mAb H12H3 at 1 µg/mL, and in PBS-Blotto with mAb H13F7 at 11 µg/mL or mAb H18C11 at 0.04 µg/mL. The same dilution series were prepared in PBS-BSA or PBS-Blotto but without mAb. These dilutions were rotated ON at 4°C. All samples were tested in duplicate.

For mAb H12H3, ELISA plates were coated ON at 4°C with 100 µL/well of 2 µg/mL VSG LiTat 1.5 in PBS and antigen-free wells served as an antigen negative control. The next day, ELISA plates were blocked at rT for 1 h with PBS-BSA and washed three times with PBST. The wells were incubated for 1 h at rT with 100 µl of the dilutions. Plates were washed three times and HRP-labelled goat Fc-specific anti-mouse IgG conjugate (Jackson), diluted 1/500 in PBST was added for 1 h (100 µL/well, rT). The colour reaction was performed as previously described for the sandwich ELISA. For the mAbs H13F7 and H18C11 the same protocol was followed, using VSG LiTat 1.3, PBS-Blotto 1% and a 1/1000 dilution of the anti-mouse conjugate.

The remaining activity (% RA) was calculated: first, for each peptide dilution, the average OD measured in the antigen-free control wells was subtracted from the average OD in the corresponding antigen containing wells, thus yielding OD_a_. Second, the corrected OD_c_ was obtained by subtracting the OD_a_ obtained in the mAb-free wells from the OD_a_ in the corresponding mAb containing wells. Finally, the % remaining activity was calculated as 100×OD_c_/OD_max_, where OD_max_ was the OD_c_ of wells receiving the peptide-free mAb dilution.

### Indirect ELISA with biotinylated peptides and anti-VSG mAbs

Nunc MaxiSorp™ plates were coated with 100 µL/well of 10 µg/mL streptavidin (NEB) in carbonate buffer (CB, 0.1 mol/L, pH 9.2). Plates were incubated ON at 4°C. After saturation with PBS-BSA (for mAb H12H3) or PBS-Blotto (for mAb H13F7) and three washes with PBST, wells were filled with 100 µL of biotinylated peptide in PBS at a concentration of 5 (only for peptide 24, C57 and C59), 1, 0.6, 0.3 and 0 µg/ml. After 1 h at rT, wells were washed three times and incubated for 1 h with 100 µL mAb (mAb H12H3 at 1.07, 0.53, 0.27 µg/mL in PBS-BSA or mAb H13F7 at 0.53 and 0.27 µg/mL in PBS-Blotto). After three washes, HRP-labelled goat anti-mouse IgG (Fcγ) conjugate was diluted 1/1000 in PBST and added at 100 µL/well for 1 h. After five washes, plates were incubated for 1 h with 100 µL/well of ABTS. The OD was read at 414 nm and corrected by subtracting the OD obtained in the peptide-free wells from the OD obtained in the corresponding peptide containing wells.

### Inhibition ELISA with biotinylated peptides and human sera

The capacity of the synthetic peptides to specifically bind antibodies in serum from HAT patients was assessed in an inhibition ELISA with nine sera from *gambiense* sleeping sickness patients and ten sera from endemic controls. ELISA plates were coated ON at 4°C with 100 µL/well of 10 µg/mL streptavidin in CB. All samples were tested in duplicate. The following day the plates were saturated with PBS-BSA (for mAb H12H3) or PBS-Blotto (for mAb H13F7). The dilutions of the biotinylated peptides were made in PBS (0.01 mol/L phosphate, 0.14 mol/L NaCl, pH 6), ranging from 20 µg/mL to 0.3 µg/mL, depending on the peptide. After three washes with PBST, the peptide dilutions were added at 100 µL/well and left for 1 h at rT. Plates were tapped dry, sealed and stored frozen at −80°C. Just before use, plates were thawed and washed (3× PBST). Wells were incubated with 100 µL of a 1/5 human serum dilution in PBS-BSA or PBS-Blotto, depending on the mAb (1 h at rT). After three washes with PBST, mAb H12H3 (in PBS-BSA) or mAb H13F7 (in PBS-Blotto) was added at a concentration ranging from 1 to 0.25 µg/mL, depending on the peptide (100 µl/well, 1 h, rT). Plates were washed three times and a 1/1000 dilution in PBST of HRP labelled goat anti-mouse IgG (Fcγ) conjugate was added at 100 µL/well (1 h, rT). Plates were washed five times and incubated for 1 h at rT with 100 µL/well of ABTS. The OD was read at 414 nm and corrected by subtracting the average OD obtained in the antigen-free wells from the average OD obtained in the corresponding antigen containing wells, thus yielding OD_c_. The percent remaining activity was calculated as 100×OD_c_/OD_max_. P-values were tested with the Wilcoxon rank test (positive versus negative) and corrected for multiple comparisons with the Bonferroni method.

### Human serum samples

Based on the results in indirect ELISA with VSG LiTat 1.5 and LiTat 1.3 (data not shown) we selected nine HAT positive (OD>1.5) and 10 endemic negative (OD<0.2) human serum samples originating from a study on detection of specific antibodies in serum and saliva in the Democratic Republic of Congo (DRC) [13].

## Results

### Peptide selection with anti-LiTat 1.5 mAb H12H3

**Figure 1:**
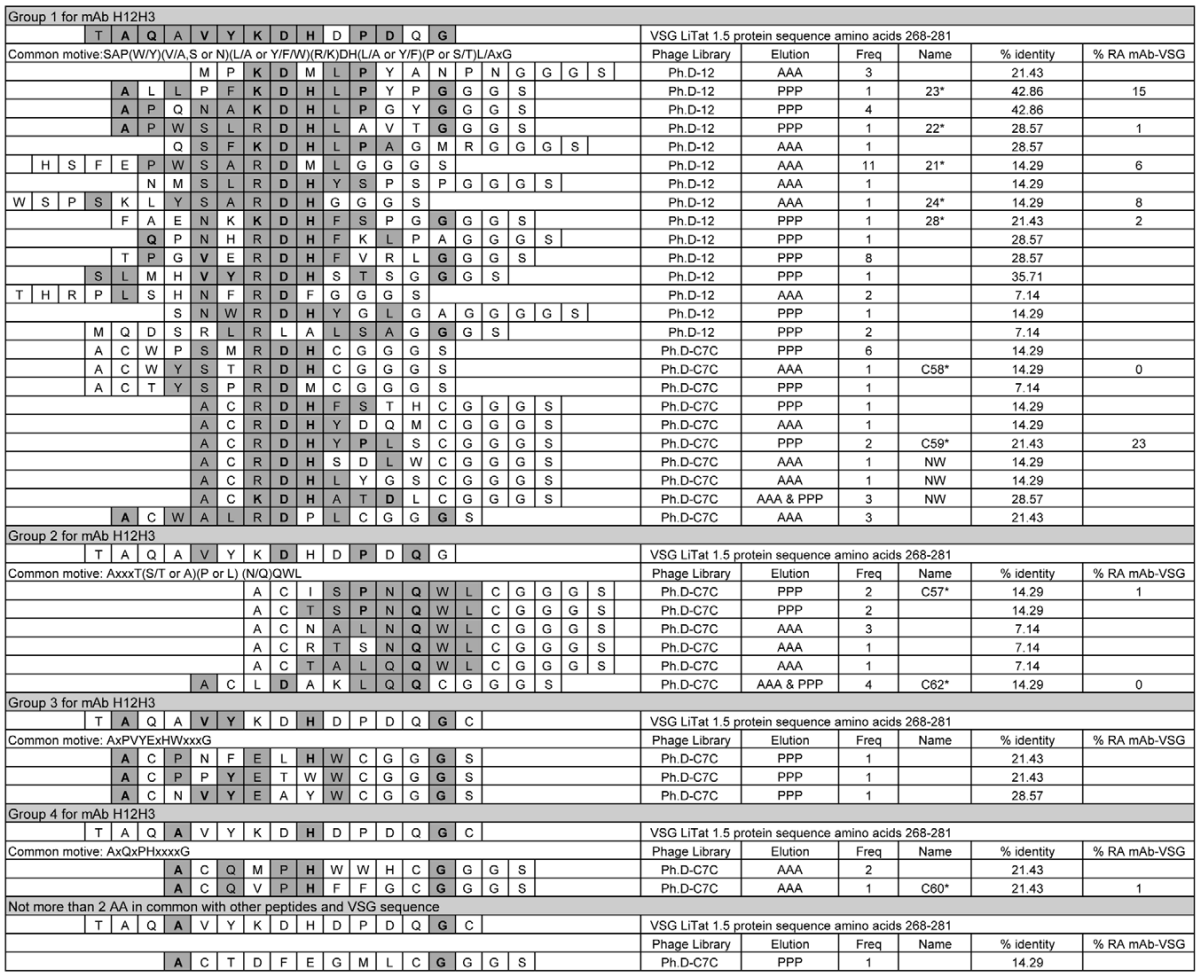
Amino acid sequences of peptides obtained by phage display with anti- VSG LiTat 1.5 mAb H12H3. The first sequence displayed is amino acid 265 to 288 of the protein sequence of VSG LiTat 1.5. Homologous amino acids are indicated in grey and amino acids that are identical to those of the VSG protein sequence are in bold and grey. All peptide sequences include the GGGS-spacer (connecting phage protein pIII and the random peptide in the original library) at the C-terminal. When expressed on the phage, the first cysteine of the C7C-peptides is preceded by alanine. AAA: eluted via antigen competition. PPP: eluted via acidification. Freq: number of times this sequence was found amongst seventy-nine selected phage clones. Name: name of the synthesised peptide.*: peptide selected for biotinylation. NW: not withheld. % identity: percentage identity of the peptide sequence with a stretch of 14 AA (AA 268 to 281) within the protein sequence of VSG LiTat 1.5. % RA mAb-VSG: percentage remaining of the mAb binding to its corresponding native VSG after inhibition by the synthetic peptide at 67 µg/ml.

Panning of the Ph.D.-12 library yielded phage clones for which median ODs in the sandwich ELISA with the homologous mAb were 1.352 (interquartile range, IQR, 1.119–1.608) for the AAA elution and 2.759 (IQR 2.384–2.943) for the PPP elution. Twenty phage clones eluted with AAA and OD>1 and twenty phage clones eluted with PPP and OD>2, were selected for amplification, cross reactivity testing and sequencing. All forty amplified phage clones reacted specifically with mAb H12H3 (median OD 1.055 with H12H3 versus 0.100 with the heterologous mAb H13F7 and H18C11), therefore excluding that the selected phage clones bound to the conserved Fc part of the mAbs. Phages obtained with the AAA elution expressed six different amino acid (AA) sequences (fig. 1). One sequence could not be read. Phages obtained with the PPP elution expressed nine different sequences.

Panning of the Ph.D.-C7C library yielded phage clones for which median ODs in the sandwich ELISA with the homologous mAb were 0.713 (IQR 0.485–2.338) for the AAA elution and 3.218 (IQR 3.138–3.324) for the PPP elution. Twenty phage clones eluted with AAA and OD>2 and twenty phage clones eluted with PPP and OD>3, were selected for amplification, cross reactivity testing and sequencing. All amplified phage clones reacted specifically with mAb H12H3 (median OD 3.106 with H12H3 versus 0.138 with the heterologous mAbs H13F7 and H18C11). Five phage clones corresponding to three sequences did not amplify well (OD with mAb H12H3<0.4) and were not withheld for further experiments. With each elution method, phages expressing twelve different amino acid sequences were obtained (fig. 1). Two of these sequences were found in both the AAA and the PPP elution, bringing the total of different C7C-sequences to twenty-two.

Amongst the thirty-seven different sequences obtained through panning with mAb H12H3, four groups of common motives could be distinguished (fig. 1). Amino acids with similar structure and characteristics were considered homologous, such as arginine (R) and lysine (K); serine (S) and threonine (T); glutamine (Q) and asparagine (N); alanine (A), valine (V), leucine (L) and isoleucine (I); phenylalanine (F), tyrosine (Y) and tryptophan (W); and aspartic acid (D) and glutamic acid (E). Group 1 with common motive SAP(W/Y)(V/A,S or N)(L/A or Y/F/W)(R/K)DH(L/A or Y/F)(P or S/T)L/AxG contained all the Ph.D.-12 sequences, and part of the Ph.D.-C7C sequences. Group 2, 3 and 4 consisted of Ph.D.-C7C sequences only and had as a common motive AxxxT(S/T or A)(P or L) (N/Q)QWL, AxPVYExHWxxxG, and AxQxPHxxxxG respectively. The sequence CTDFEGMLC did not have more than two AA in common with one of the other sequences and is displayed separately.

Homology between peptides and the protein sequence of VSG LiTat 1.5 [GenBank HQ662603] was found within AA 268 to 281 of the protein sequence (maximum 42.86% or 6/14 identical AA, fig. 1), in the variable N-terminal domain of VSG LiTat 1.5.

Out of the thirty-seven obtained sequences, ten peptides were synthesised. All ten synthetic peptides strongly inhibited the binding of mAb H12H3 to native VSG LiTat 1.5 in a dose dependent manner (<25% remaining activity at a peptide concentration of 67 µg/ml) (fig. 1) and were resynthesised with a C-terminal lysine-biotin. Peptides selected with mAb H13F7 and H18C11 (see below) did not inhibit binding of H12H3 to VSG LiTat 1.5, (data not shown). All biotinylated peptides were recognised by mAb H12H3 in an indirect ELISA. A peptide concentration of >10 µg/mL was necessary to obtain an OD>0.5 with peptides C57 and C59 while 5 µg/mL for peptide 24 and concentrations ranging from 0.3 to 0.6 µg/mL for the other seven peptides, were sufficient to obtain an OD>1 (data not shown).

### Peptide selection with anti-LiTat 1.3 mAb H13F7

**Figure 2:**
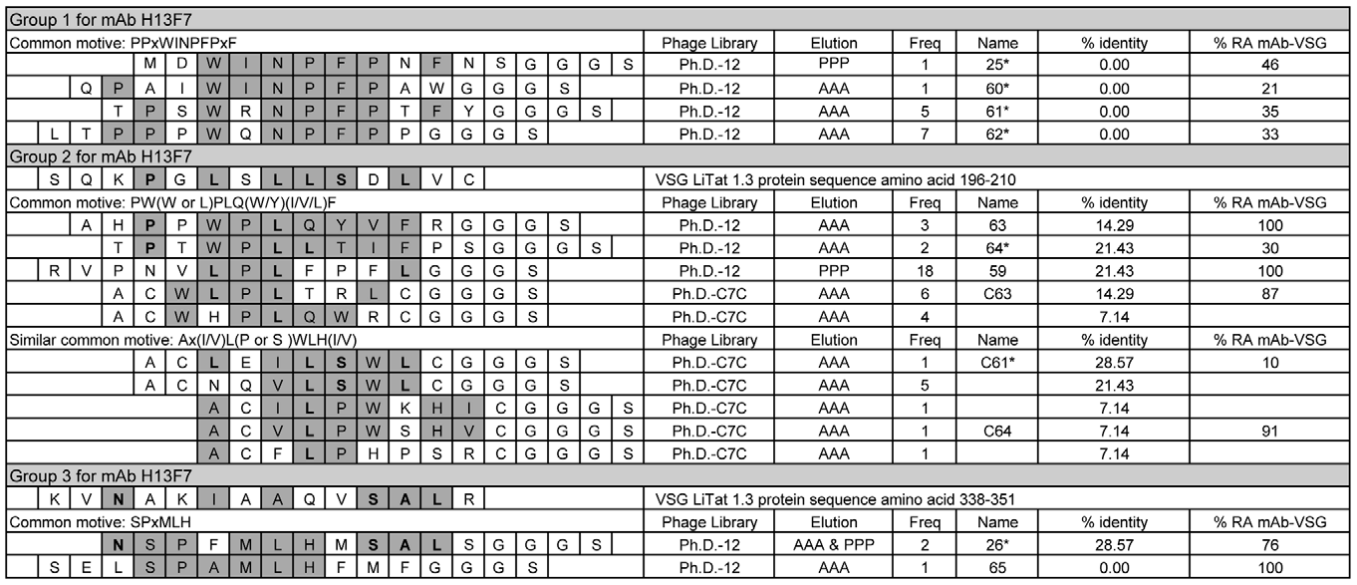
Amino acid sequences of peptides obtained by phage display with anti-VSG LiTat 1.3 mAb H13F7. The amino acid stretches 196 to210 and 338 to 351 of the VSG LiTat 1.3 protein are displayed in group 2 and 3 respectively. Homologous amino acids are indicated in grey and amino acids that are identical to those of the VSG protein sequence are in bold and grey. All peptide sequences include the GGGS-spacer (connecting phage protein pIII and the random peptide in the original library) at the C-terminal. When expressed on the phage, the first cysteine of the C7C-peptides is preceded by alanine. AAA: eluted via antigen competition. PPP: eluted via acidification. Freq: number of times this sequence was found amongst fifty-nine selected phage clones. Name: name of the synthesised peptide.*: peptide selected for biotinylation. % identity: percentage identity of the peptide sequence with a stretch of 14 AA within the protein sequence of VSG LiTat 1.3. % RA mAb-VSG: percentage remaining of the mAb binding to its corresponding native VSG after inhibition by the synthetic peptide at 67 µg/ml.

Panning of the Ph.D.-12 library yielded phage clones for which median ODs in the sandwich ELISA with the homologous mAb were 3.219 (IQR 3.000–3.350) for the AAA elution and 0.148 (IQR 0.123–2.999) for the PPP elution. Per elution method, twenty phage clones with OD>2 were selected. All forty amplified clones reacted specifically with mAb H13F7 (median OD 3.022 with H13F7 versus 0.113 with the heterologous mAbs H12H3 and H18C11); this excludes that the selected phage clones bound to the conserved Fc part of the mAbs. Phages obtained with the AAA elution, expressed seven different amino acid sequences. Phages obtained with the PPP elution, expressed three different sequences. One sequence was found in both the AAA and the PPP elution, thus bringing the total of different sequences to nine (fig. 2).

Panning of the Ph.D.-C7C library yielded phage clones for which median ODs in the sandwich ELISA with the homologous mAb were 1.481 (IQR 0.739–1.869) with the AAA eluted phage clones and 0.107 (IQR 0.087–0.122) with the PPP eluted phage clones. None of the PPP eluted clones were withheld. Twenty phage clones of the AAA elution with OD>1 were amplified. All reacted specifically with mAb H13F7 (median OD 1.942 with H13F7 versus 0.106 with the heterologous mAbs H12H3 and H18C11). Seven different amino acid sequences were expressed (fig. 2).

Amongst the sixteen different sequences obtained with mAb H13F7, three groups of common motives could be distinguished (fig. 2). Group 1 with common motive PPxWINPFPxF contained only 12-mer sequences. Group 2 contained some of the 12-mer and all of the 7-mer sequences and had as common motive PW(W or L)PLQ(W/Y)(I/V/L)F or, with “WPL” in reverse order, Ax(I/V)L(P or S )WLH(I/V). Peptide 59 and peptide C63 share a common motive, but in reverse order (F/W)LPL. Group 3 consisted of two sequences with common motive SPxMLH. Alignment of these sequences with the protein sequence of VSG LiTat 1.3 [GenBank AJ304413], only gave results for group 2 and one sequence of group 3. These had maximum 28.57% identical AA (4/14) within respectively AA stretch 196 to 210 and AA stretch 338 to 351 of VSG LiTat 1.3 (fig. 2). The motive W(AA 291)P(AA 290)L(AA 292)L(AA 234)T(AA 230) of peptide 64 could be mapped onto the three-dimensional VSG LiTat 1.3 protein structure, in the N-terminal domain (results not shown).

Out of the sixteen sequences selected with mAb H13F7, twelve peptides were synthesised. Six synthetic peptides strongly inhibited the binding of the mAb to the native VSG LiTat 1.3 (<50% remaining activity at a peptide concentration of 67 µg/ml), and one peptide was a weaker inhibitor (76% remaining activity) (fig. 2). These seven peptides were resynthesised with a C-terminal lysine-biotin and their reactivity with mAb H13F7 was assessed by indirect ELISA. All biotinylated peptides had an OD>1 with mAb H13F7 at a concentration of 0.6 to 0.3 µg/mL peptide (data not shown).

### Peptide selection with anti-LiTat 1.3 mAb H18C11

Panning of the Ph.D.-12 library yielded phage clones for which median ODs in the sandwich ELISA with the homologous mAb were 0.124 (IQR 0.109–0.185) for the AAA elution and 0.130 (IQR 0.108–0.148) for the PPP elution. None of the clones from the Ph.D-C7C-library gave a sufficiently high OD to be amplified. Twenty AAA eluted clones of the Ph.D.-12-library with OD>0.5 were amplified. All twenty clones reacted specifically with mAb H18C11 (median OD 3.271 with H18C11 versus 0.106 with the heterologous mAbs H12H3 and H13F7). Only one amino acid sequence was expressed: SHSTPYYWKGYI. We could not identify any homology between this peptide and the protein sequence of VSG LiTat 1.3. The corresponding synthetic peptide did not react with mAb H18C11 in indirect ELISA (data not shown) and did not inhibit the binding of mAb H18C11 to native VSG LiTat 1.3 and was therefore not withheld for further experiments.

### Assessment of the diagnostic potential of the biotinylated peptides for HAT by inhibition ELISA with human sera

**Figure 3:**
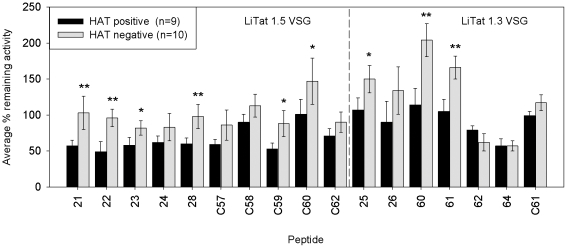
Average percent remaining activity of the mAb binding to the biotinylated synthetic peptides. The ability of human serum antibodies to bind to the mimotopic peptides selected with anti-VSG monoclonal antibodies was assessed by means of an inhibition ELISA. *: *p*<0.05 and **: *p*<0.01. Compared to the ten HAT negative sera, the nine HAT positive sera significantly inhibited the binding of anti-LiTat 1.5 mAb H12H3 to peptide 21, 22, 23, 28, C59 and C60 and the binding of anti-LiTat 1.3 mAb H13F7 to peptides 25, 60 and 61.

The diagnostic potential of the biotinylated peptides was assessed in an inhibition ELISA with human sera from nine *gambiense* HAT patients and ten negative controls. Compared to the HAT negative sera, the HAT positive sera significantly inhibited binding of mAb H12H3 to peptide 23, C59 and C60 (*p*<0.05) and 21, 22, and 28 (*p*<0.01) (fig. 3). Five of these peptides belong to common motive group 1, peptide C60 belongs to group 4 (fig. 1). The HAT positive sera also significantly inhibited binding of mAb H13F7 to peptide 25 (*p*<0.05) and peptides 60 and 61 (*p*<0.01) (fig. 3). All three peptides belong to common motive group 1 (fig. 2).

## Discussion

By means of phage display technology we successfully identified peptides that mimic epitopes on the native trypanosomal variant surface glycoproteins LiTat 1.5 and LiTat 1.3 of *T.b. gambiense*. These mimotopic peptides were recognised by the monoclonal anti-VSG antibodies H12H3 and H13F7 that were used for panning, and might have potential for the diagnosis of human African trypanosomiasis.

Our results indicate that a linear region in the protein sequence of VSG LiTat 1.5 was identified. This region is localised in the N-terminal domain of the VSG near the surface of the trypanosome and is therefore a candidate for further testing as a synthetic, linear peptide. Antibodies specific to linear, continuous epitopes on protein antigens typically contact three to four critical amino acids over a six residue segment [16]. The peptide sequences selected with mAb H12H3 had up to six amino acids (% identity 42.86) in common with the variable N-terminal domain (AA 268 to 281) of VSG LiTat 1.5. Glycine from the GGGS-spacer, inserted between the peptide sequence and the pIII phage protein, was part of this common motive. It is possible to define the exact residues in the peptide sequences that are essential for binding with the antibody by alanine scanning mutagenesis and recreate the epitope of each monoclonal. A recent example has been the identification of a linear epitope on the VP1 protein of foot-and mouth disease virus by Yang *et al.* [29] by screening a 12-mer phage display library with a mAb.

Contrary to VSG LiTat 1.5, we suspect the epitope of VSG LiTat 1.3 to be discontinuous. Alignment of the peptide sequences with the protein sequence of VSG LiTat 1.3 located the common motives in different parts of the protein sequence with a maximum % identity of only 28.57. Also, when the VSG was (partly) denatured, the OD in ELISA with this mAb dropped. Many protein epitopes are discontinuous and comprise critical binding residues that are distant in the primary sequence but close in the folded native tertiary protein structure. Indeed, WPLLT, the motive of peptide 64 was mapped onto the three-dimensional VSG LiTat 1.3 protein structure, near the surface of the trypanosome. As “WPL” or “LPW” is part of the common motive in the peptide sequences of group 2 (fig. 2) it is possible that the discontinuous epitope of mAb H13F7 is localised in this region.

All 12-mer peptides that strongly inhibited the binding of the mAbs to the VSG contain one or more proline residues. Proline limits the flexibility of the peptide and may therefore favour the forming of the mAb-peptide complex [30]. The 7-mer peptides are already constrained by two flanking cysteines, which may account for the fact that the sequence of four of the best cyclic inhibitors contains no proline.

Cortese [31] reports that many mAbs fail to select specific peptides. Due to the limitation of library complexity it is often impossible to isolate peptides of high affinity and there is no general rule applicable as to what type of library suits a certain application [18]. In our study mAb H18C11 selected specific peptides, but only with the 12-mer library. Additionally, all phage clones selected with this mAb expressed the same peptide sequence. It is possible that one phage clone overgrew other, higher-affinity phage clones, during amplification. The peptide selected with mAb H18C11 and some of the synthetic peptides selected with mAb H12H3 and mAb H13F7 failed to react with the corresponding mAb in indirect ELISA. It has been described before that phage-born peptides can lose their ability to bind the target molecule when synthesised chemically [17]. Furthermore the conformation of peptides in binding assays may differ from the presentation on the phage. Although direct coating was only successful for some peptides (data not shown), we successfully demonstrated the capacity of several of these peptides in solution to inhibit the binding of their mAb to the corresponding VSG. Based on these results we selected some peptides for resynthesis and biotinylation. The biotinylated peptides were bound to streptavidin, which was coated onto the ELISA plate, this improved the peptide presentation to such an extent that all of the biotinylated peptides were able to bind their corresponding mAb.

Although the aim of our study was to identify mimotopes for antibody detection in human serum, we opted to perform the panning with mouse monoclonal antibodies. Indeed, sera from sleeping sickness patients contain an important fraction of trypanosome unrelated antibodies as a consequence of polyclonal B cell stimulation [32], [33]. Therefore, the risk to select mimotopes unrelated to sleeping sickness by applying human sera for the panning is considerable, unless only the trypanosome specific antibody fraction of these sera is used. Moreover, it was demonstrated that mAbs identified peptide mimotopes similar to those selected with pooled sera of typhus patients [21]. Also, mAb H13F7 was able to cause lysis of trypanosomes of VAT LiTat 1.3 in the immuno-trypanolysis test, which demonstrates that this mAb recognises VSG epitopes, exposed on living bloodstream trypanosomes, similar to those recognised by human sleeping sickness sera [3]. Finally, we chose to perform the screening with mAbs that bind to different epitopes on the VSGs to increase the chance that the selected mimotopes would bind different antibodies in the polyclonal patient sera.

Human sleeping sickness sera inhibited the binding of anti-LiTat 1.5 mAb H12H3 to peptides 21, 22, 23, 28, C59 and C60 and the binding of anti-LiTat 1.3 mAb H13F7 to peptides 25, 60 and 61, auguring for their value as diagnostic antigens. Not all peptides were equally well recognised by human sera. It is possible that some peptides react weakly with positive sera in spite of their specificity for immunodominant regions, which can be explained if these peptides mimic only part of the structure of the corresponding region on the antigen [20]. This may be the reason why the mimotopes corresponding to the linear region in the protein sequence of VSG LiTat 1.5 that interacts with the mAb seem to be more easily recognised by human antibodies than the mimotopes of the discontinuous LiTat 1.3 epitope, where a correct conformational presentation is crucial. The mouse and human immune system may as well react with different vigour to certain epitopes or recognise different principal epitopes. The fraction of antibodies in HAT sera that bind the same epitope as the mAbs may therefore be relatively small. By using an inhibition ELISA, low serum dilutions could be applied, maximising the reaction with the peptides, but avoiding the non-specific reactions often observed with HAT sera at low dilutions. Unexpectedly, with several peptides, addition of human serum in the inhibition ELISA resulted in higher ODs than those obtained with the serum-free control wells. This observation might be explained if human serum contains a substance that changes the conformation of a peptide in a way that facilitates binding to the mAb.

With this study, we aimed at delivering the proof of principle that mimotopes for VSGs can be selected from a peptide phage display library making use of mAbs. However, the study has some limitations. Firstly, by making use of only three mAbs, it is likely that other diagnostic mimotopes have been missed. Secondly, no affinity measurements *e.g.* via surface plasmon resonance, have been performed. Considering the polyclonal character of antibodies in patients' sera and the inherent differences in antibody response between individual patients, we opted to assess only the diagnostic potential of the selected peptides by means of ELISA.

In conclusion, we successfully demonstrated that polyclonal antibodies in human sleeping sickness sera recognise mimotopes of VSG LiTat 1.3 and 1.5, indicating diagnostic potential of peptides selected with monoclonal antibodies. Still, replacement of the native *T.b. gambiense* LiTat 1.3 and LiTat 1.5 VSGs in the currently existing diagnostic formats might not be straightforward. It should be preceded by confirming the diagnostic potential of the selected peptides, or variations and combinations thereof, on larger panels of HAT positive and negative sera, and, if needed, peptide sequence optimisation.
